# Effects of Surface Charge Distribution and Electrolyte Ions on the Nonlinear Spectra of Model Solid–Water Interfaces

**DOI:** 10.3390/molecules29163758

**Published:** 2024-08-08

**Authors:** Konstantin S. Smirnov

**Affiliations:** Univ. Lille, CNRS, UMR 8516 - LASIRe - Laboratoire Avancé de Spectroscopie pour les Interactions la Réactivité et l’Environnement, F-59000 Lille, France; konstantin.smirnov@univ-lille.fr

**Keywords:** solid–water interface, structure, nonlinear spectra, molecular dynamics simulations

## Abstract

Molecular dynamics simulations of model charged solid/water interfaces were carried out to provide insight about the relationship between the second-order nonlinear susceptibility $\chi^{\left(2\right)}$ and the structure of the interfacial water layer. The results of the calculations reveal that the density fluctuations of water extend to about 12 Å from the surface regardless of the system, while the orientational ordering of molecules is long-ranged and is sensitive to the presence of electrolytes. The charge localization on the surface was found to affect only the high-frequency part of the Im[$\chi^{\left(2\right)}$] spectrum, and the addition of salt has very little effect on the spectrum of the first water layer. For solid/neat water interfaces, the spectroscopically active part of the liquid phase has a thickness largely exceeding the region of density fluctuations, and this long-ranged nonlinear activity is mediated by the electric field of the molecules. The electrolyte ions and their hydration shells act in a destructive way on the molecular field. This effect, combined with the screening of the surface charge by ions, drastically reduces the thickness of the spectroscopic diffuse layer. There is an electrolyte concentration at which the nonlinear response of the diffuse layer is suppressed and the $\chi^{\left(2\right)}$ spectrum of the interface essentially coincides with that of the first water layer.

## 1. Introduction

Solid/water interfaces are ubiquitous and play important roles in a plethora of natural phenomena and industrial processes [1,2]. As examples, one can mention the heterogeneous chemistry of the atmosphere and geochemistry, heterogeneous catalysis, tribology, semiconductor manufacturing, and biomedical applications. Despite the importance of interfaces, the knowledge of the details of the processes in a tiny region close to the surface at the atomic level is far from being complete. Unveiling the microscopic mechanisms of interfacial phenomena necessitates the use of surface-specific techniques [3], among which, nonlinear vibrational spectroscopy takes a particular place. The surface specificity of nonlinear spectroscopy results from the fact that the bulk centrosymmetry is broken at the interface, which makes the second-order nonlinear susceptibility $\chi^{\left(2\right)}$ nonzero. The method, which includes second-harmonic generation (SHG) and vibrational sum-frequency generation (VSFG) spectroscopy, is sensitive, non-invasive, and suitable for buried surfaces, and it does not require vacuum conditions [4,5]. In addition to the information about chemical species present at the interface, heterodyne-detected sum-frequency generation spectroscopy (HD-VSFG) also makes it possible to obtain structural information about the interfacial region [6,7,8]. All of these features have led to the widespread use of SHG and VSFG spectroscopies in the investigation of solid/water interfaces, and of mineral/water ones, in particular [2,9,10].

The structure and dynamics of (charged) water interfaces are commonly discussed on the basis of an electrical double layer (EDL) model [11,12]. According to the model, a first layer of water molecules with solvated ions is referred to as a Stern layer, while a more distant part of loosely bound water is called a diffuse layer (DL). The definition of a Stern layer is intimately related to the presence of electrolyte ions in the aqueous phase, and computer simulation studies have shown that the Stern layer is essentially identical to the first layer of water bound to the surface: the bonded interfacial layer (BIL) [13,14,15]. Henceforth, the paper uses BIL as an acronym to designate the first layer of interfacial water, regardless of the presence of ions in the liquid phase.

Most of surface-specific phenomena occur in the BIL. However, the nonlinear response of the interface comes from both the BIL and the DL, and this fact hinders setting up a molecular picture of processes in the topmost water layer. Consequently, much effort has been applied to disentangle the spectral signatures of the BIL and the DL in the experimental HD-VSFG spectra of solid/water interfaces [13,16,17,18,19]. A common outcome of these studies is that the DL spectrum is mainly determined by the third-order nonlinear susceptibility of bulk water and by the electric potential across the layer. Consequently, the shape of the DL spectrum has been found to be to a significant extent common to all charged solid/liquid surfaces. Based on this finding, the separation of the BIL and DL contributions has provided considerable insight about the structure and bonding of water next to silica surfaces [16,17,18,19].

Experimental research on solid/water interfaces by nonlinear spectroscopy has been supported by modeling studies that have employed both DFT-based and classical molecular dynamics (MD) simulations. A great deal of such investigations have focused on mineral/water interfaces, and the outcomes of these works was recently reviewed by Borguet and co-workers [10,20]. The results of the computations have shed light on the relation of the nonlinear spectra with the structure of the interfacial region. Thus, the shape and intensity of OH bands in HD-VSFG spectra have been linked to the surface morphology and chemistry, to the composition of the liquid phase, and to the electric potential across the interface [14,15,21,22,23,24,25,26,27,28]. MD simulations have shown that the BIL spectrum is sensitive to the surface structure and chemistry [15,21,25,27], while the DL spectrum, in agreement with the experimental findings, is mainly determined by the third-order nonlinear susceptibility of bulk water and by hte electric potential in the layer [15,21,22,26]. Electrolyte ions were observed to affect mainly the DL spectrum [15,21]. However, the typical ion concentrations used in modeling, especially in the DFT-based MD simulations, were often too high compared to the concentrations used in experiments.

The interpretation of VSFG spectra, even supported by the results of MD simulations, is often hampered by the fact that real surfaces are not flat, and thus, the surface morphology can affect the spectra in a surface-specific manner. The presence of active surface sites with different strengths combined with surface corrugation further complicates understanding the spectra–structure relationship. For instance, it was found that a formally hydrophilic silica surface can have the spectral signature typical of a hydrophobic one [29]. Thus, the question of to what extent the VSFG spectra of aqueous interfaces reflect the characteristics of the interfacial water is far from being completely understood.

The objective of the present study was twofold. Firstly, the work was aimed at investigating nonlinear spectra of the BIL and the DL regions of solid/water interfaces as a function of the charge distribution on the surface. Secondly, since the aqueous phase always contains solvated ions, the work addressed the effect of ions, particularly at a low concentration, on the nonlinear spectra of the solid/water interface. To attain the goals, the structural characteristics and nonlinear spectra of a water layer in contact with a model charged solid surface were obtained in classical molecular dynamics computer simulations. The considered model surface is flat and is characterized by a uniform or localized distribution of charges. The aqueous phase is either a neat water or NaCl solution with a concentration of 0.1 M or 0.5 M. These concentrations are in the range of the values used in experiments, and the former is close to the lower limit of concentrations that still allow statistically reliable modeling of ions’ effects on the nonlinear response at a reasonable computational cost. The study was carried out bearing mineral/water interfaces in mind, and as the normal pH of water is typically higher than the point of zero charge of many mineral surfaces [30,31,32], these are negatively charged. Consequently, the primary emphasis of the work was placed on the behavior of water in contact with a negatively charged surface. Raw data for positively charged solid/neat water interfaces are provided in Supplementary Materials.

## 2. Models and Computations

### 2.1. Simulation Setup

**Figure 1:**
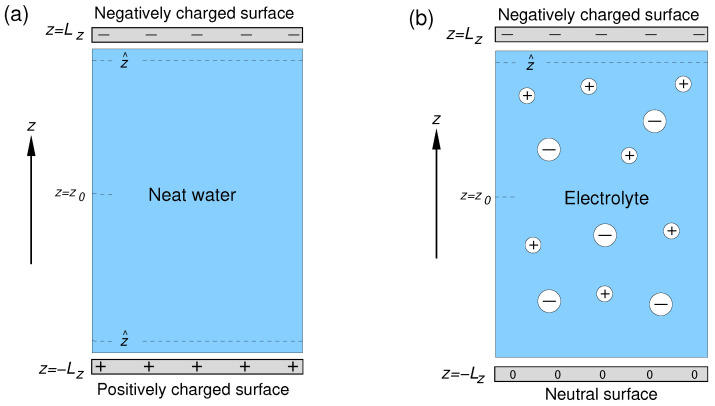
Setups of simulation cell used in simulations of the solid/neat water interface (**a**) and solid/electrolyte solution interface (**b**); $\hat{z}$ and $z_{0}$ denote the *z*-coordinate of the interface and of the mid-plane between the surfaces, respectively.

The setups of the simulation cells used in the study are shown in Figure 1. The aqueous phase was sandwiched between two solid surfaces lying in the xy Cartesian plane and separated by a distance $2L_{Z}=135.2$ Å. The distance was chosen so that the water density in the central part of the layer coincided with the bulk water density. Each surface was represented by a layer of 200 atoms forming a 2D fcc lattice with a lattice parameter of 2.17 Å. The origin of the *z*-axis was placed in the midpoint between the surfaces. The simulation cells had dimensions of 21.7×21.7×200.0 Å along the *x*, *y*, and *z* Cartesian axes, respectively, and 3D periodic boundary conditions were applied to the cells.

The two surfaces of the solid/neat water system bore a charge of the same magnitude but of opposite sign (Figure 1a). For solid/electrolyte systems, the surface at $z=L_{Z}$ was charged negatively, whereas that at $z=-L_{Z}$ was neutral (Figure 1b). The required number of sodium ions was added to the liquid phase of the solid/electrolyte systems to compensate for the negative surface charge.

### 2.2. Interatomic Potentials

Interatomic interactions in the systems were described by a sum of the Lennard–Jones (12–6) and Coulomb potentials. Water molecules were represented with the SPCFw model [33], and the atoms of the solid surface were described with Lennard–Jones parameters for the carbon atom of the OPLS-AA force field [34]. Parameters for the Na^+^ and Cl^−^ ions were taken from ref. [35]. The Lorentz–Berthelot combining rules were used to obtain the Lennard–Jones parameters for unlike atoms.

Charged surfaces possessed a charge of $5\;|e^{-}|$, which corresponds to a surface charge density of $1.06\;|e^{-}|$/nm^2^. Each surface atom of the systems with a uniform charge distribution carried a charge of $0.025\;|e^{-}|$. For a surface with localized charges, a charge of $0.25\;|e^{-}|$ was assigned to 20 atoms that were randomly chosen on each surface, which gives a surface density of 4.25 ${\mathrm{nm}}^{-2}$ for the charged sites. Atoms bearing a charge were chosen under the constraint that for every charged atom, none of its nearest neighbors could carry a charge. Just for comparison with real surfaces, the charge density of $-1\;|e^{-}|$/nm^2^ for a silica surface corresponds to an alkaline aqueous phase with pH>9 [30,32]. Table 1 summarizes the characteristics of the solid/water interfaces studied in this work and gives the acronyms used in this paper to refer to the systems.

### 2.3. MD Simulations

The classical equations of motion were integrated using the velocity form of the Verlet algorithm with an integration time-step of 0.5 fs. The real-space cut-off radius for the short-range and electrostatic interactions was equal to 10 Å. The discontinuity of the short-range energy and force at the cut-off distance was treated with a shifted-force technique. The long-range electrostatic interactions were handled using a damped shifted-force modification of the Wolf method [36,37] with a damping parameter of 0.2 Å^−1^ [37]. The temperature in the MD runs in an NVT statistical ensemble was controlled via a chain of Nosé–Hoover thermostats.

For the UQ@H_2_O system, simulations started from a 10 ns equilibration NVT run at a temperature of 293 K. Then, four configurations that were randomly selected from the last 2 ns part of the trajectory and were spaced by at least 200 ps were used as the starting configurations for four series of 25 production runs. For the three other systems (Table 1), four initial structures with different distributions of either charges or electrolyte ions were generated and equilibrated in 10 ns NVT MD runs for T=293 K. The final configurations of these runs were then used as starting points for four series of 25 production runs. For all the systems, each production run consisted of a 50 ps equilibration period in the NVT ensemble that was followed by a 50 ps NVE stage, where the coordinates and velocities of atoms were saved each 4 fs during the last 40 ps. The final configuration of the previous run was used as the starting configuration of the next run, and the atomic velocities in each simulation were newly chosen from the Maxwell–Boltzmann distribution. Such a simulation protocol was aimed at an enhanced sampling of the phase space.

### 2.4. Structural Characteristics

The structural organization of the interfacial water in the direction perpendicular to the surface was characterized by a profile of the relative water density $\rho {\left(z\right)}^{*}=\rho \left(z\right)/\rho_{0}$, where $\rho_{0}$ is the density of bulk liquid water. A *z*-dependent net orientation of water molecules with respect to the surface normal was obtained by computing 〈cosθ〉 as a function of the *z*-coordinate, where θ is the angle between the molecular dipole and the *z*-axis; MD simulations of bulk water for T=293 K yielded 〈cosθ〉=0 within the statistical error. In a similar way, the distribution of Na^+^ and Cl^−^ ions in the interfacial region was visualized by computing their profiles along the normal to the surface. The intermolecular H-bonds in the systems were identified and analyzed with the use of geometric criteria [38].

The orientation of H_2_O molecules with respect to the *z*-axis was described by a 2D distribution of the conditional probability density $P_{Z}\left(u_{1},u_{2}\right)$:

(1) $$P_{Z}\left(u_{1},u_{2}\right)=\frac{1}{N_{Z}}\langle \sum_{i\in Z}\sum_{k\in i}\delta \left(u_{1}-\cos\phi_{1}^{\left(i\right)}\right)\cdot \delta \left(u_{2}-\cos\phi_{2}^{\left(i\right)}\right)\rangle ,$$

where δ() stands for the Dirac delta-function, and $\cos\phi_{k}^{\left(i\right)}$ is the cosine of the angle between the *k*-th OH bond vector of molecule *i* and the *z*-axis. The distribution is computed for molecules with a CoM lying in the region Z, and the normalization factor $N_{Z}$ in (1) is given by

(2) $$\int_{-1}^{1}P_{Z}\left(u_{1},u_{2}\right)\;du_{1}du_{2}=1.$$

In the following, the work discusses a relative probability density ${\tilde{P}}_{Z}\left(u_{1},u_{2}\right)=P_{Z}\left(u_{1},u_{2}\right)/$$P_{\infty }\left(u_{1},u_{2}\right)$, where $P_{\infty }\left(u_{1},u_{2}\right)$ is the probability density distribution (1) in an isotropic medium [39].

### 2.5. Nonlinear Spectra Computation

Nonlinear spectra of the interfacial water layer were computed with the time correlation function approach [40]. According to the approach, the pqr component of the resonant part (*R*) of the frequency-dependent nonlinear second-order susceptibility tensor $\chi^{\left(2\right)}\left(\omega \right)$ is given by

(3) $$\chi_{pqr}^{\left(2,R\right)}\left(\omega \right)=\frac{i\omega }{k_{B}T}\int_{0}^{\infty }dt\;e^{ı\omega t}\langle M_{r}\left(0\right)\cdot A_{pq}\left(t\right)\rangle ,$$

where $M_{r}$ and $A_{pq}$ are the *r* and pq elements of the system dipole M and polarizability A, respectively. The present work focuses on the spectrum obtained as the average of the spectra of the xxz and yyz elements of $\chi^{\left(2,R\right)}$. This spectrum is denoted with a ssp subscript throughout the paper, as it can be related to a VSFG spectrum recorded with the ssp polarization combination (the *s*- and *p*-polarized visible and infrared pulses, respectively, and the *s*-polarized sum-frequency radiation). Additional details on the computation of the second-order susceptibility can be found in ref. [24].

### 2.6. Analysis of the $\chi^{\left(2,R\right)}$ Spectra

Interpretation of the nonlinear spectra of solid/water interfaces commonly uses the assumption that the second-order nonlinear susceptibility $\chi^{\left(2\right)}$ of the interfacial region can be expressed as a sum of second-order hyperpolarizabilities $\beta_{i}$ of individual molecules [40,41,42]

(4) $$\chi^{\left(2\right)}=\sum_{i}\beta_{i}.$$

A perturbation theory expression for the pqr element of the β quantity reads [4]

(5) $$\beta_{pqr}\left(\omega \right)\approx \sum_{k}\left(\frac{\partial \alpha_{pq}}{\partial Q_{k}}\right)\left(\frac{\partial \mu_{r}}{\partial Q_{k}}\right)\cdot \frac{1}{\left(\omega_{k}-\omega \right)-ı\Gamma_{k}},$$

where the sum runs over vibrational modes *k*. In (5), $\alpha_{pq}$ and $\mu_{r}$ are the pq and *r* components of the molecular polarizability α and the dipole μ, and $Q_{k}$, $\omega_{k}$, and $\Gamma_{k}$ denote the normal coordinate, frequency, and damping factor of the *k*-th vibrational mode, respectively. From (5) one readily obtains that

(6) Im[βpqr](ω)≈∑k∂αpq∂Qk∂μr∂Qk·Γk(ωk−ω)2+Γk2.

Therefore, the spectrum of the imaginary part of $\beta_{pqr}$ is a sum of Lorentzian functions weighted by the product of the derivatives of the dipole and polarizability components in the normal modes, and the sign of the product in a particular mode determines the sign of the contribution from the mode to the Im[$\beta_{pqr}$] spectrum. As the direction of the transition dipole is related to the molecular frame, the analysis of the sign allows the establishment of a qualitative relation between the sign of the features in the spectrum and the orientation of the molecule (see Appendix A for details). Although the above consideration deals with an isolated H_2_O molecule, a similar analysis can be applied to an ensemble of molecules. In this way, the position, magnitude, and sign of the spectral bands in the Im[$\chi^{\left(2\right)}$] spectrum, which are accessible in HD-VSFG experiments, can shed light on the strength of intermolecular interactions and on the net orientation of molecules.

Further insight into the origins of the nonlinear polarizability of the interfacial water layer was obtained by separating its nonlinear response into two contributions. Within the polarizable point dipole model [40,43] used in the study, the dipole moment M of the system can be written as a sum of two terms:

(7) $$M=M^{0}+M^{I}=\sum_{i}\mu_{i}^{0}+\sum_{i}\alpha_{i}E_{i},$$

where $M^{0}$ is given by a sum of permanent dipole moments $\mu_{i}^{0}$ and reflects the net orientation of the molecules, while $M^{I}$ is the sum of the induced dipole moments and depends on the polarizability $\alpha_{i}$ and on the electric field $E_{i}$ acting on the polarizable dipoles. Then, by substituting $M_{r}$ with $M_{r}^{0}$ and $M_{r}^{I}$ in (3), one gets two contributions to $\chi_{pqr}^{\left(2\right)}$. The first one stems from the lack of the inversion symmetry due to the presence of the surface, whereas the second contribution is due to the polarization of water by the electric field in the interfacial region. In what follows, these two contributions are referred to as orientational and induced, respectively.

The induced contribution $\chi_{ind}^{\left(2\right)}$ can be readily recognized as the $\chi^{\left(3\right)}$-term, which is given by the following expression [22,44]:

(8) $$\chi_{ind}^{\left(2\right)}=\chi_{B}^{\left(3\right)}\int_{-\infty }^{\hat{z}}E\left(z\right)dz=-\chi_{B}^{\left(3\right)}\left(\varphi \left(\hat{z}\right)-\varphi \left(-\infty \right)\right),$$

where $\chi_{B}^{\left(3\right)}$ is the third-order nonlinear susceptibility of bulk water, E(z) is the electric field along the normal to the surface, and φ() denotes the electrostatic potential. The integration in (8) runs from a point at z=−∞ in the bulk region of the liquid phase to the interface at $z=\hat{z}$, which implies that the *z*-axis is directed towards the surface. In this way, (8) meets the convention commonly used in the interpretation of the results of VSFG studies of solid/liquid interfaces.

## 3. Results and Discussion

### 3.1. Structural Characteristics

**Figure 2:**
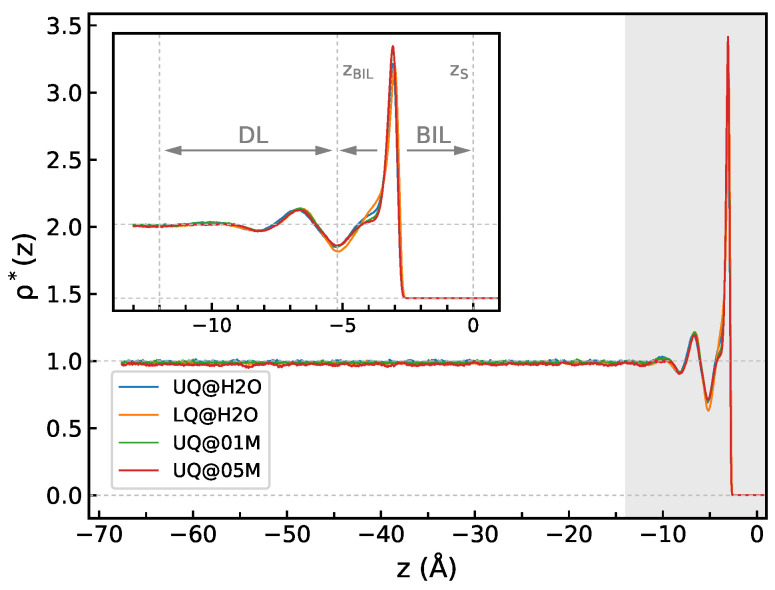
The *z*-profile of water density $\rho {\left(z\right)}^{*}$ for solid/water interfaces. The inset shows a zoom of the shaded area and indicates limits of the bonded interfacial layer (BIL) and the diffuse layer (DL); see text detail. The origin of the *z*-axis is at the position of the surface atoms: $z_{S}$.

Figure 2 shows the $\rho^{*}\left(z\right)$ density profiles computed for the solid/water interfaces. The profiles reflect a layered structure of water, with density fluctuations extending to a distance of about 12 Å from the surface, and the density in the region with z<−12 Å is essentially equal to its bulk value (Figure 2). The density profiles look very much similar for all the systems, and the thickness of BIL, defined as the position of the first minimum of $\rho^{*}\left(z\right)$ at $z=z_{BIL}$, is virtually independent of the type of charge distribution or of the presence of electrolyte ions in the liquid phase. Following previous studies [14,24], the region with $-12\;Å<z<z_{BIL}$ covering the density fluctuations can be ascribed to a diffuse layer, which therefore has a thickness of ca. 7 Å. However, as will be shown below, the spectroscopically active part of the interfacial layer does not necessarily coincide with the structurally perturbed water region.

**Figure 3:**
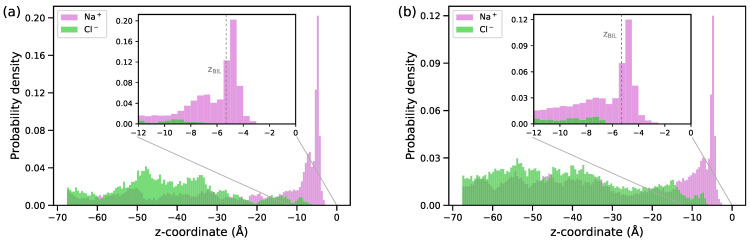
Distribution of ions along the direction perpendicular to the surface for NaCl solutions with concentrations of 0.1 M (**a**) and 0.5 M (**b**). The origin of the *z*-axis is at the position of the surface atoms; the vertical dashed line in the inset plot shows the *z*-coordinate of the BIL (see Figure 2).

Figure 3 displays the distribution of ions in the interfacial region for the two electrolyte concentrations. A high probability of finding the Na^+^ ions in the BIL reflects the attraction of the cations by the negative charge of the surface while the layer is depleted of anions. The surface density of the Na^+^ ions in the layer is 0.4 ${\mathrm{nm}}^{-2}$ and 0.6 ${\mathrm{nm}}^{-2}$ at NaCl concentrations of 0.1 M and 0.5 M, respectively, and both these values are notably smaller than the charge surface density of $1.06\;|e^{-}|$/nm^2^. No overcharging of the BIL occurs.

**Figure 4:**
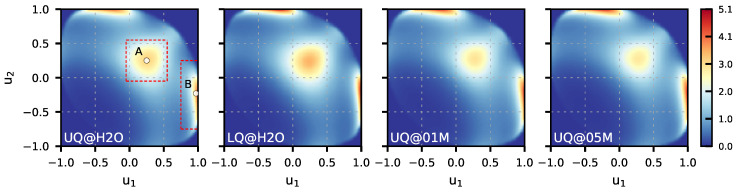
Maps of conditional probability density ${\tilde{P}}_{BIL}\left(u_{1},u_{2}\right)$ (1) of solid/water interfaces. Circles labeled “A” and “B” in the UQ@H_2_O map indicate two preferred orientations of water molecules, which are sketched in Figure 5. The red dashed rectangles show the area used to estimate the water population in the two orientations reported in Table 2.

**Figure 5:**
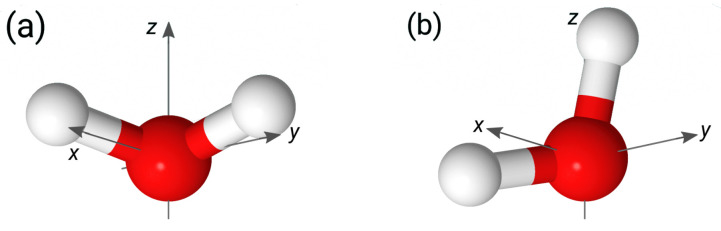
Preferred orientations of water molecules in the BIL of the systems. Panels (**a**,**b**) display the orientations marked in Figure 4 with circles and labelled as A and B, respectively. The *z*-axis in the panels is directed to the surface.

Figure 4 presents the 2D maps of the ${\tilde{P}}_{Z}\left(u_{1},u_{2}\right)$ probability computed for the BILs of the systems. The maps reveal two preferred orientations of water molecules in the layer. The first orientation, labeled by “A” in Figure 4 (UQ@H2O panel), is characterized by two OH bonds directed to the surface ($u_{1}\approx u_{2}\approx 0.25$), while the second orientation, denoted as “B”, has one bond oriented towards the surface ($u_{1}\approx 1.0$) and the second one pointing to the liquid phase ($-0.75<u_{2}<0$). These two orientations are illustrated in Figure 5. The orientation “A” can reflect molecules involved in intermolecular bonding and/or molecules bound to the surface by two H atoms. Concerning the orientation “B”, one can reasonably assume that such an orientation reflects H_2_O molecules interacting with the surface by one of the OH bonds. As the pattern in the maps in Figure 4 essentially remains the same, the orientational ordering of the molecules in the BIL is not perturbed by either the charge localization or the presence of ions in the liquid phase. Table 2 gives estimates for the population of molecules with two orientations and shows that these are roughly equal to each other with variations depending on the system.

**Figure 6:**
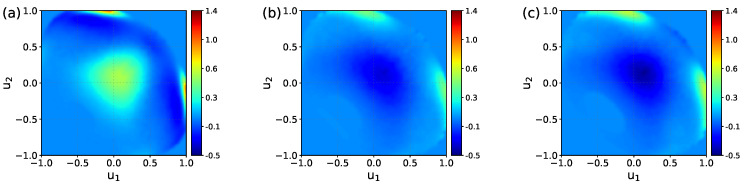
Difference of the ${\tilde{P}}_{BIL}\left(u_{1},u_{2}\right)$ maps in Figure 4: LQ@H_2_O–UQ@H_2_O map (**a**), UQ@01M–UQ@H_2_O map (**b**), and UQ@05M–UQ@H_2_O map (**c**).

To examine the effect of the charge distribution/ions on the water ordering in the BIL in more detail, one can consider a difference between the ${\tilde{P}}_{BIL}\left(u_{1},u_{2}\right)$ maps. Such difference maps are shown in Figure 6, with the map for the UQ@H_2_O system taken as the reference. One sees that the charge localization on the surface increases the probability of finding both types of molecular orientations in the BIL. On the other hand, the presence of ions in the liquid phase suppresses molecules with the orientation of type “A” while promoting the orientation of type “B”. This analysis is confirmed by data in Table 2.

**Figure 7:**
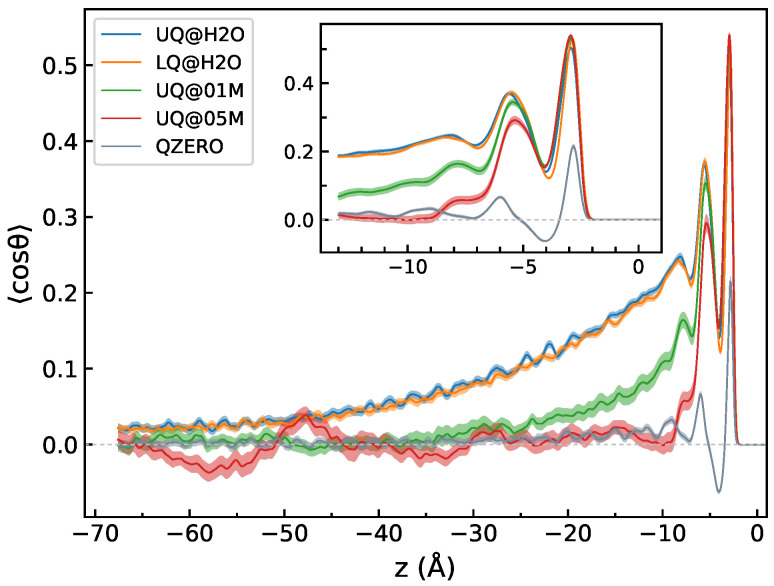
The *z*-profile of 〈cosθ〉, where cosθ is the angle between the molecular dipole and the *z*-axis. The *z*-profile labeled “QZERO” was computed for a neutral surface/neat water interface. The inset plot shows a zoom of the region of the water density fluctuations, cf. Figure 2. The origin of the *z*-axis is at the position of the surface atoms. Colored areas indicate standard deviations.

Figure 7 displays the *z*-profiles of 〈cosθ〉, where θ is the angle between the molecular dipole and the *z*-axis. For the sake of comparison, Figure 7 also shows such a profile computed for neat water in contact with a neutral surface. The behavior of the 〈cosθ〉 profiles in the zone z>−12 Å is similar for all charged interfaces, with bumps in the profiles related to the density fluctuations in that region (Figure 2). Beyond the DL region, i.e., in the bulk part with z<−12 Å, the 〈cosθ〉 profile reveals a dependence on the composition of the liquid phase, in contrast to the density. In this region, the mean value of cosθ decays rather slowly with the distance from the surface for the solid/neat water charged interfaces compared to the neutral surface. The presence of electrolyte ions in the liquid phase markedly reduces the decay length, and for the 0.5 M electrolyte solution, the 〈cosθ〉 quantity oscillates around zero for distances greater than 12 Å. A similar long-ranged alignment of water molecules was obtained by Dreier et al. in MD simulations of model membrane/water interfaces [45].

Table 3 presents the statistics of intermolecular H-bonds in the bulk, DL, and BIL regions of the systems in terms of the total number of H-bonds ($N_{T}$) and the number of H-bonds parallel to the surface ($N_{\|}$). These in-plane bonds were identified according to ref. [46] as hydrogen bonds with |cosψ|<0.5, where ψ is the angle of the donor–acceptor vector with the surface normal. For bulk liquid water, calculations give $N_{T}=3.50\pm 0.01$ and $N_{\|}=1.76\pm 0.02$, and the ratio $N_{T}/N_{\|}$ agrees with an analytical estimate $N_{T}/N_{\|}=2$ for an isotropic medium.

The H-bonds statistics in the bulk part and the DL show that both the number of H-bonds and the $N_{T}/N_{\|}$ ratio are close to the values characteristic of bulk liquid water. However, a common trend is that the presence of electrolyte ions results in a decrease in the number of H-bonds in those regions. The decrease in $N_{T}$ with the increase in the electrolyte concentration was attributed to the formation of a hydration shell of ions that destroys the intermolecular H-bonding [26]. On the other hand, molecules in the BIL form fewer intermolecular hydrogen bonds, and almost two-thirds of these bonds, compared with approximately half in bulk and the DL, have the donor–acceptor vector close to the surface plane. One can also notice a greater number of in-plane H-bonds in the BIL of the LQ@H_2_O interface compared to the three other systems (Table 3). This is in line with a larger probability of finding molecules with the “A” orientation in the BIL of this system (Figure 6a). Indeed, molecules involved in in-plane H-bonding are likely have OH bonds oriented so that $u_{1,2}\approx 0.25$. Regarding the other interfaces, no definite conclusions can be drawn about the change in the number of in-plane H-bonds, as the $N_{\|}$ values are close to each other within the statistical uncertainties.

It should be mentioned that a more detailed analysis of H-bond network is possible [47]. For the water–vapor interface such an analysis shows that the reduction of number of intermolecular H-bonds in the first molecular layer is mostly due to molecules most close to the interface and that the decrease is mainly caused by the paucity of intralayer bonding. The analysis scheme is worth to be employed in future studies of solid/water interfaces.

The results presented above allow us to draw the following picture of the structural organization of the aqueous phase in the proximity of the model solid surfaces. The interfacial water has a layered structure that extends to a distance of ∼12 Å from the surface, and the first water layer (BIL) has a thickness of about 5.3 Å regardless of the system. The water density beyond the structurally perturbed region coincides with its bulk value, and the thickness of the diffuse layer can be estimated as ca. 7 Å. Water molecules in the BIL are organized in such a way that part of them have two OH bonds that are roughly parallel to the surface (Figure 5a), while the other molecules direct one of their OH bonds towards the surface and the second one points to the liquid phase (Figure 5b). In contrast to the density, the net orientation of the molecules at the solid/neat water charged interface remains perturbed far beyond the distance of 12 Å from the surface. The presence of the electrolyte ions markedly shortens the depth of the orientational perturbation. The BIL molecules form, on average, 2.7 intermolecular H-bonds, of which approximately 1.9 bonds are in-plane H-bonds, which can be ascribed to bonding between the molecules lying parallel to the surface. As suggested by the analysis of Figure 6, the charge localization increases the probability of finding both of the orientations in the BIL, whereas the solvation of electrolyte ions destroys the in-plane H-bonding network.

### 3.2. Nonlinear Spectra

Given the information about the structure of the interfacial water layer, let us turn to the analysis of the spectra of the second-order nonlinear susceptibility $\chi_{ssp}^{\left(2\right)}$. Making use of the discussion in Section 2.6 and Appendix A, the two OH stretching vibrations of water molecules with the orientation “A” (Figure 5a) are expected to give a positive signal in the Im$\left[\chi_{ssp}^{\left(2\right)}\right]$ spectrum of the BIL because $u_{1}$ and $u_{2}$ are both positive. However, as the *u* values are small, the intensity of the signal is likely to be rather low (The derivative $\left(\partial \mu_{z}/\partial Q_{k}\right)$ can be approximated as $|(\partial \mu /\partial Q_{k})|\cdot u$ with *u* being cosine of the angle of OH bond with the *z*-axis). The vibrations of molecules with the orientation “B” (Figure 5b) should yield two spectral signals with opposite signs at frequencies that depend on the strength of the interaction of the OH bonds with their surroundings. The charge of the surface atoms $q_{S}$ (Table 1) is smaller than the oxygen charge in the SPCFw water model ($q_{O}=-0.82\;\left|e^{-}\right|$), which suggests that the OH bond directed to the surface is less perturbed than the bond involved in H-bonding with its neighboring molecules. As the “B” molecules can likely be related to the molecules that have one bond interacting with the surface, one may then expect that the molecules will give a positive high-frequency and a negative low-frequency contribution to the Im$\left[\chi_{ssp}^{\left(2\right)}\right]$ spectrum of the BIL, with the intensity of the former bigger than that of latter because $|u_{1}\left|>\right|u_{2}|$ and $u_{2}$ varies over a wide range (Figure 4). The sum of the signals by the molecules with the two orientations then yields the *orientational* contribution to the nonlinear spectrum of the BIL.

**Figure 8:**
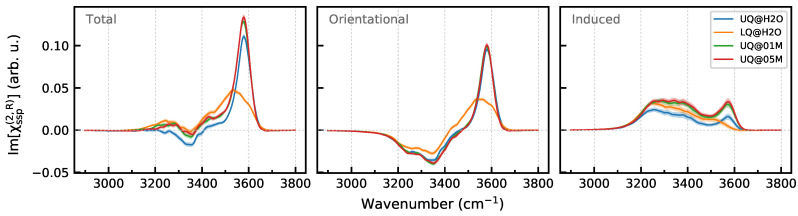
Breakdown of Im[$\chi_{ssp}^{\left(2\right)}$] spectra of the BIL of the solid/water interfaces. Panels in the row display the total spectrum and its orientational and induced components. Colored areas indicate standard deviations.

Figure 8 displays the Im$\left[\chi_{ssp}^{\left(2\right)}\right]$ spectra of the BIL and their breakdown into the orientational and induced components. As anticipated by the analysis above, the orientational component has a high-frequency positive band at 3580 ${\mathrm{cm}}^{-1}$ and a low-frequency negative band at 3350 ${\mathrm{cm}}^{-1}$, with the former having a higher intensity. For the surfaces with the uniform charge distribution, the orientational component is virtually insensitive to the composition of the liquid phase. The charge localization on the surface results in a red shift and broadening of the high-frequency band. This effect can be attributed to the surface heterogeneity. As the surface now possesses charged sites and neutral patches, the high-frequency positive band of the LQ@H_2_O system probably has two components. The first one is at lower wavenumbers and results from molecules interacting with the sites that bear negative charges with an order of magnitude greater than the atoms of the uniformly charged surface, whereas the second component is at higher wavenumbers and comes from molecules adsorbed at the neutral patches.

The induced component of the Im$\left[\chi_{ssp}^{\left(2\right)}\right]$ spectra of the BIL is positive and has a similar shape for all systems, with the only exception being the LQ@H_2_O one, for which the high-frequency peak at 3580 ${\mathrm{cm}}^{-1}$ disappears in the spectrum (Figure 8). The peak at this wavenumber is due to weakly perturbed OH oscillators, such as molecules interacting with the neutral patches, and as the patches do not yield any polarization, no signal appears in the induced spectrum at the high frequencies. At the same time, the signal of the OH bonds interacting with the charged sites is shifted to lower wavenumbers, where it overlaps with the induced spectrum of other OH bonds. The presence of ions in the aqueous phase leads to an increase in the intensity of the induced component of the BIL induced spectrum. Making use of (8), one can calculate the difference of the electrostatic potential $\Delta \varphi_{21}=\varphi \left(z_{2}\right)-\varphi \left(z_{1}\right)$ between points 2 and 1 as

(9) $$\Delta \varphi_{21}=-\int_{z_{1}}^{z_{2}}E_{z}\left(z\right)dz,$$

where $E_{z}\left(\right)$ is the component of the electric field along the *z*-axis (Appendix B). The differences of the potential $\Delta \varphi_{BIL}=\varphi \left(z_{S}\right)-\Delta \varphi \left(z_{BIL}\right)$ across the BILs computed in this way are $\Delta \varphi_{BIL}\approx -600$ meV and $\Delta \varphi_{BIL}\approx -850$ meV for the solid/neat water and solid/electrolyte interfaces, respectively. The higher magnitude of $\Delta \varphi_{BIL}$ for the latter systems explains the higher intensity of the induced component in the corresponding spectra in Figure 8.

Concluding the discussion of the BIL spectra, it is important to stress that signals of molecules with the “A” orientation interfere with those of “B” molecules. As a result, a simple analysis of the total spectrum would suggest that the BIL contains only molecules with the “B” orientation, and thus, the presence of molecules with the “A” orientation in the layer would be overlooked.

**Figure 9:**
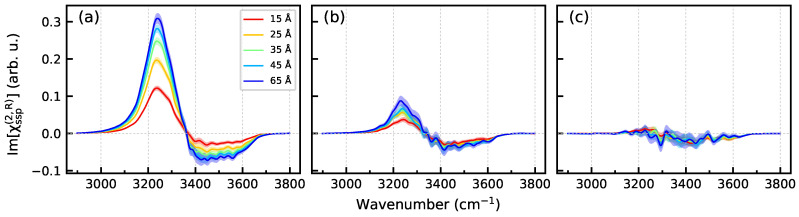
Im[$\chi_{ssp}^{\left(2\right)}$] spectra of the region from $z_{BIL}$ to $z=z_{0}$ (Figure 1) as a function of the distance from the surface. (**a**)—UQ@H_2_O system, (**b**)—UQ@01M system, and (**c**)—UQ@05M system. The *z* values in the legends in panel (**a**) stand for the distance from the surface. Colored areas indicate standard deviations.

Figure 9 presents Im$\left[\chi_{ssp}^{\left(2\right)}\right]$ spectra of a region extending from $z=z_{BIL}$ to the mid-plane between the two surfaces at $z=z_{0}$ (Figure 1) as a function of the distance from the surface. The spectra of the UQ@H_2_O system clearly show that the spectroscopically active part of the liquid phase, the spectroscopic diffuse layer (SDL), extends far beyond the DL defined as the structurally perturbed part of the water between the BIL and the bulk region (Figure 2). The SDL thickness for the solid/neat water interface can be estimated to be at least 30 Å. The electrolyte ions in the liquid phase markedly reduce the thickness of the SDL so that the spectra of the UQ@05M system do not show any *z*-dependence within the statistical uncertainty, and the SDL thickness is virtually that of the DL. The behavior of SDL spectra in Figure 9 correlates with the *z*-dependence of the 〈cosθ〉 profiles in Figure 7.

**Figure 10:**
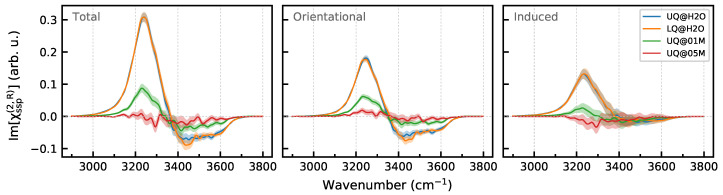
Breakdown of Im[$\chi_{ssp}^{\left(2\right)}$] spectra of the DL of the solid/water interfaces. Panels in the row display the total spectrum and its orientational and induced components. Colored areas indicate standard deviations.

Figure 10 shows the Im$\left[\chi_{ssp}^{\left(2\right)}\right]$ spectra computed for a region from $z=z_{BIL}$ to $z=z_{0}$ and the breakdown of the spectra into the orientational and induced components. One sees that the charge localization has essentially no effect on the spectrum of the layer. This result could be expected, as water molecules of the layer do not directly “see” the surface, and thus, the nonlinear response is impacted by the surface only in an indirect way. Contrarily, the presence of ions in the liquid phase, already at the concentration of 0.1 M, notably affects the spectrum. The intensities of both the orientational and induced components decrease, and further growth of the electrolyte concentration to 0.5 M almost completely suppresses the spectral signal of the diffuse layer.

The effect of ions on the orientational component can be related to the chaotropic effect due to the formation of the ions’ solvation shell, which breaks intermolecular interactions and brings structural heterogeneity to the layer. Table 3 shows that the ions reduce the number of intermolecular H-bonds in the DL without affecting the $N_{T}/N_{\|}$ ratio, which remains close to two within the statistical uncertainty.

**Figure 11:**
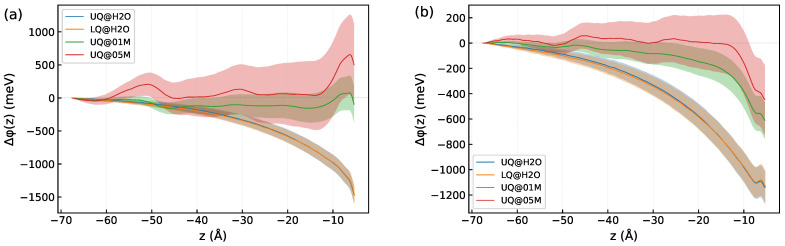
The *z*-profiles of the difference Δφ(z) of the electrostatic potential (9) in the diffuse layer of the systems: (**a**)—total potential and (**b**)—dipole potential. Colore areas indicate standard deviations. The origin of the *z*-axis is placed at the position of the surface atoms.

The influence of ions on the induced component of the DL spectrum deserves more detailed consideration. According to (8), the magnitude of the induced component is determined by the $\chi^{\left(3\right)}$ nonlinear susceptibility, which is a bulk property, and by the electrostatic potential difference across the diffuse layer. Figure 11 shows the *z*-profiles of the electrostatic potential difference Δφ(z) computed by (9). In these computations, the $z_{1}$ integration limit was set to $z_{0}$ (mid-plane between the surfaces, Figure 1), and $z_{2}$ ran from $z=z_{0}$ to $z=z_{BIL}$. The total potential displayed in Figure 11a is due to the electric field caused by charges of the surface and electrolyte ions and by the field of molecular dipoles, while the dipole potential presented in Figure 11b comes from the dipole field only (see Appendix B for detail). One sees that the way the charge is distributed on the surface has no effect on either the total or dipole potential, which accounts for the insensitivity of the induced spectrum to the type of charge distribution on the surface (Figure 10). It is noteworthy that the total potential in the solid/neat water interfaces is rather long-ranged, and the behavior results from a slowly decaying dipole potential (Figure 11b). The total potential for these systems can be nicely fitted by an exponential function with a decay length of 19 Å.

The presence of ions in the liquid phase drastically changes the behavior of the electric potential in the DL. Since the surface charge term remains the same for the interfaces with both neat water and electrolyte solution, the changes in the behavior of the potential are entirely due to (i) screening of the surface charge by cations in the BIL and (ii) the destructive effect of ions with their solvation shells on the dipole field in the DL. The combination of these two factors reduces the nonlinear activity of the spectroscopic diffuse layer, which becomes almost inactive at the 0.5 M concentration of NaCl (Figure 10). It should be noted that the screening of the surface charge likely has a limited impact on the SDL spectra in the present case, as the density of ions in the BIL is notably less than the surface charge density. On the other hand, the destructive effect of the ions and their hydration shells can be mediated by water molecules by imposing a specific H-bonding pattern beyond the hydration shell. The predominance of either factor for a particular system may depend on such characteristics as the pH of the liquid phase, the surface chemistry and morphology, and the concentration and nature of the ions. For instance, the reorganization of the interfacial water by salt ions has been suggested to be a main reason of the change of the nonlinear response of a negatively charged silica/water interface [28].

Unfortunately, large statistical fluctuations of the potential hamper an estimation of the decay length of Δφ(z) for the solid/electrolyte systems. However, the *z*-dependence of the dipole potential in Figure 11b implies a decrease in the decay length upon increasing the electrolyte concentration, which agrees with the predictions by the mean-field theories of the EDL. The behavior of Δφ(z) is in line with the *z*-profile of 〈cosθ〉 in Figure 7. Both quantities are long-ranged for the solid/neat water interfaces, and they decay with distance more rapidly when ions are present in the liquid phase.

**Figure 12:**
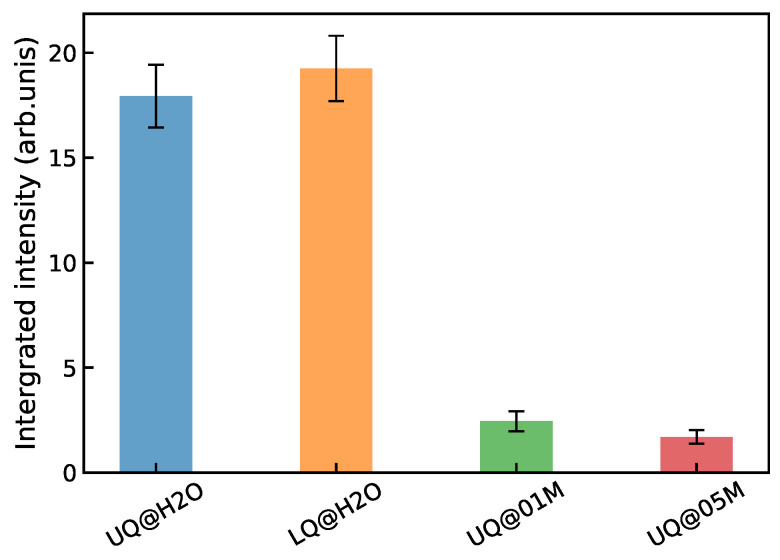
Integrated intensity of the $|\chi_{ssp}^{\left(2\right)}{|}^{2}$ spectra of the solid/water interfaces.

Figure 12 shows the integrated intensity of the $|\chi_{ssp}^{\left(2\right)}{|}^{2}$ spectrum for the systems. The intensity of the solid/neat water interfaces is practically insensitive to the way in which the charge is distributed on the surface. On the other hand, the presence of electrolyte ions in the liquid phase, even at a small concentration, reduces the intensity of the nonlinear signal by about an order of magnitude. A similar trend of the VSFG intensity with the ion concentration has been found experimentally for silica/water interfaces [17,19,28,48,49,50]. The intensity was measured to have a maximum at concentrations of about $10^{-4}$ M, and then it decreased and remained nearly constant in the concentration range 0.1 M to 1 M. It has been argued that the nonlinear response mostly comes from the $\chi^{\left(2\right)}$ susceptibility of the BIL in this concentration range [48,49]. The results presented above explain the intensity behavior shown in Figure 12 as a partial or complete suppressing of the SDL’s contribution to the nonlinear spectrum of the solid/electrolyte interface, and this rationalization supports the conclusion of the experimental studies.

## 4. Conclusions

An extensive molecular dynamics study of model charged solid/water interfaces with different types of surface charge distributions and concentrations of electrolyte ions sheds light on the relationship between spectra of the second-order nonlinear susceptibility $\chi^{\left(2\right)}$ of the interfacial water layer and its structural characteristics. The results of the calculations reveal that the density fluctuations of water extend to about 12 Å from the surface regardless of the type of surface charge distribution and the concentration of the electrolyte. In contrast to the water density, the net orientation of molecules was obtained to be more long-ranged, and it is highly sensitive to the presence of electrolyte ions.

The calculations show that the charge localization on the surface affects only a high-frequency part of the Im[$\chi^{\left(2\right)}$] spectrum of the BIL. In the range of studied concentrations, the addition of salt was found to have very little effect on the BIL spectra: it mainly affected the intensity of the induced component. The spectroscopically active part of solid/neat water interfaces has a larger thickness than the region of water density fluctuations. This long-ranged nonlinear activity is mediated by a dipole electric field of water molecules. The electrolyte ions and their hydration shells act in a destructive way on the molecular field by bringing heterogeneity into the structural organization of the aqueous phase. This effect, combined with the screening of the surface charge by ions in the BIL, drastically reduces the thickness of the spectroscopic diffuse layer. There is an electrolyte concentration at which the SDL nonlinear response is completely suppressed and the $\chi^{\left(2\right)}$ spectrum of interface essentially coincides with that of the BIL. This outcome supports the conclusions of experimental studies on the dependence of the VSFG intensity on the electrolyte concentration.

Despite the apparent simplicity of the studied model systems, extracting the information about the structure and bonding in the interfacial region from the nonlinear spectra is strongly hampered by the fact that the total signal is a sum of the signed contributions of different spectral components of the BIL and SDL regions. For real surfaces, a further complication comes from their chemical and structural heterogeneity. Consequently, molecular dynamics simulations, both classical and DFT-based, are of great help for disentangling the surface-specific nonlinear spectra of solid/water interfaces. Yet, the simulations have to use as realistic of a surface model as possible, and an enhanced sampling procedure is highly advisable for coming to definite conclusions.

## Figures and Tables

**Table 1:** Characteristics of the systems studied in this work.

Interface	Surface Charge ^1^	$q_{S}$ ^2^ ($|e^{-}|$)	No. H_2_O ^3^	No. Na^+^/Cl^− 4^	Acronym
Solid/neat water	Uniform	−0.025	2058	–	UQ@H_2_O
Solid/neat water	Localized	−0.25	2058	–	LQ@H_2_O
Solid/electrolyte	Uniform	−0.025	2045	9/4	UQ@01M
Solid/electrolyte	Uniform	−0.025	2013	25/20	UQ@05M

^1^ Type of surface charge distribution. ^2^ Charge of charged sites. ^3^ Number of water molecules. ^4^ Number of cations/anions.

**Table 2:** Estimates of population (in %) of molecules with the orientations “A” and “B” in BIL of the model solid/water interfaces.

System	UQ@H2O	LQ@H2O	UQ@01M	UQ@05M
Orientation A	24.2	26.1	23.1	22.8
Orientation B	22.6	20.5	25.2	26.0

**Table 3:** Number of intermolecular H-bonds per water molecule in the bulk, DL, and BIL regions of the systems; the values in parentheses are statistical uncertainties. $N_{T}$ and $N_{\|}$ denote the total number of H-bonds and the number of H-bonds parallel to the surface, respectively.

System	Bulk			DL			BIL	
	$N_{T}$	$N_{\|}$		$N_{T}$	$N_{\|}$		$N_{T}$	$N_{\|}$
UQ@H_2_O	3.50 (0.01)	1.74 (0.03)		3.51 (0.06)	1.71 (0.07)		2.73 (0.08)	1.97 (0.11)
LQ@H_2_O	3.50 (0.02)	1.75 (0.03)		3.49 (0.06)	1.70 (0.07)		2.77 (0.09)	2.09 (0.12)
UQ@01M	3.46 (0.01)	1.73 (0.03)		3.45 (0.06)	1.68 (0.07)		2.63 (0.08)	1.89 (0.11)
UQ@05M	3.35 (0.01)	1.67 (0.03)		3.48 (0.06)	1.67 (0.07)		2.63 (0.08)	1.91 (0.11)

**Figure A1:**
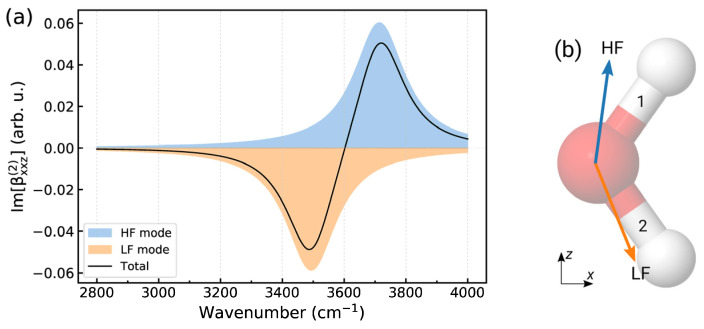
(**a**) Model Im[$\beta_{xxz}$] spectrum of water molecule. Colored shaded areas show the contributions from the high-frequency (HF) and low-frequency (LF) vibrational modes, and the total spectrum is drawn using a black solid line. (**b**) Orientation of water molecules used in the computation of the spectra in panel (**a**). Light blue and orange arrows indicate the (∂μ/∂Q) vectors in the HF and LF modes, respectively.

## Data Availability

The raw data supporting the conclusions of this article will be made available by the authors on request.

## Supplementary Materials

The following supporting information can be downloaded at: https://www.mdpi.com/article/10.3390/molecules29163758/s1, Figure S1: Breakdown of Im[$\chi_{ssp}^{\left(2\right)}$] spectra for negatively charged solid/water interfaces; Figure S2: Snapshots of BIL for the negatively and positively charged UQ@H2O interfaces; Figures S3–S5: Structural characteristics and nonlinear spectra of positively charged solid/neat water interfaces.

## Abbreviations

The following abbreviations are used in this manuscript:

VSFG	Vibrational sum-frequency generation spectroscopy
SHG	Second harmonic generation spectroscopy
HD-VSFG	Heterodyne-detected VSFG
MD	Molecular dynamics
EDL	Electrical double layer
BIL	Bonded interfacial layer
DL	Diffuse layer
SDL	Spectroscopic diffuse layer

## Appendix A. Structural Information from Im[χ^(2)^] Spectra

To illustrate the usage of (6) in the structural analysis, Figure A1a displays a calculated Im[$\beta_{xxz}$] spectrum of the water molecule shown in Figure A1b. The calculations used the results of the normal-mode analysis of SPCFw water, and to mimic intermolecular interactions, the force constant of bond 2 was taken to be 10 % smaller than that of bond 1. Such a toy model can represent a molecule of water/vapor interface, with bond 1 pointing to the vacuum and bond 2 directed to the liquid phase. The dipole and polarizability derivatives in (6) were computed by numerical differentiation of the quantities obtained with the models described in ref. [24]. The computed ray spectrum was convoluted with a Lorentzian function with $\Gamma_{k}=100$ ${\mathrm{cm}}^{-1}$; see (6).

The calculated $\left(\partial \alpha_{xx}/\partial Q\right)$ derivatives in the high-frequency and low-frequency OH stretching modes are both positive, whereas the $\left(\partial \mu_{z}/\partial Q\right)$ quantities have a positive and negative sign, respectively, and the (∂μ/∂Q) vectors are approximately directed along the OH bonds (Figure A1b). From the analysis of the positions and the signs of the bands in the spectrum, it can then be deduced that the free (strong) OH bond is oriented in the positive direction of the *z*-axis, while bond 2, which is weakened by the interactions with the liquid phase, points in the negative direction.

## Appendix B. Computation of Electric Field

An electric field $E(r_{i})$ at a point *i* defined by the coordinate vector $r_{i}$ is given by

(A1) $$E\left(r_{i}\right)=E_{q}\left(r_{i}\right)+E_{\mu }\left(r_{i}\right)$$

with $E_{q}$ and $E_{\mu }$ being electric fields due to $N_{q}$ charges *q* and $N_{\mu }$ dipoles μ, respectively. In the work, the charge field is due to atoms of the solid surface and electrolyte ions, while the dipole field results from water molecules (polarizable dipoles). Expressions for the charge and dipole fields read

(A2) $$\begin{array}{cc} E_{q}\left(r_{i}\right) & =\sum_{j}^{N_{q}}\frac{q_{j}}{r_{ij}^{2}}\;u_{ij} \end{array}$$

(A3) $$\begin{array}{cc} E_{\mu }\left(r_{i}\right) & =\sum_{k}^{N_{\mu }}{\hat{T}}_{ik}\;\mu_{k}, \end{array}$$

where $u_{ij}=r_{ij}/r_{ij}$ and $r_{ij}=r_{i}-r_{j}$, and dipole–dipole interaction tensor ${\hat{T}}_{ik}$ has the following form:

(A4) $${\hat{T}}_{ik}=\frac{1}{r_{ik}^{3}}\left(3\;u_{ik}⊗u_{ik}-I\right).$$

In (A4), I is the identity matrix and ⊗ denotes the outer product. The field (A1) is then fed to (7) for computing the molecular dipoles, and the *z*-components of the fields (A1) and (A3) are used in (8) for the computation of the total and dipole electrostatic potentials, respectively.
