# Supplementary material for: Effects of Surface Charge Distribution and Electrolyte Ions on the Nonlinear Spectra of Model Solid–Water Interfaces
Source: Molecules. 2024 Aug 8;29(16):3758. doi: 10.3390/molecules29163758 (PMC11356812; doi:10.3390/molecules29163758)
Supplement: Supplementary file 1 [file molecules-29-03758-s001.zip › supplementary.pdf]

Supplementary Materials: Effects of Surface Charge Distribution and Electrolyte Ions on the Nonlinear Spectra of Model Solid–Water Interfaces

Konstantin S. Smirnov

1. Breakdown of Im[$\chi^{(2)}_{ssp}$] spectra for negatively charged solid/water interfaces.

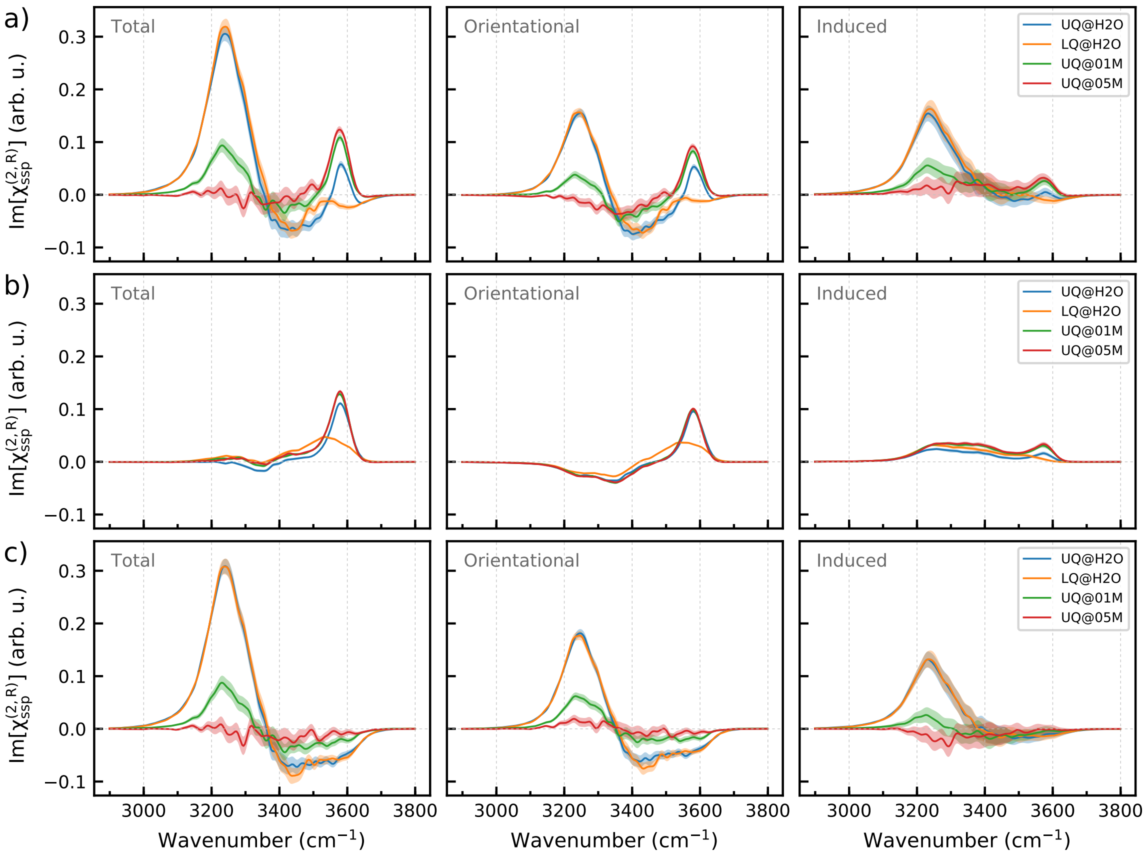

Figure S1. Breakdown of Im[$\chi^{(2)}_{ssp}$] spectra for the negatively charged solid/water interfaces. (a) – spectra of a region from $z = z_{BIL}$ to $z = z_0$ (Fig. 1 of main article), (b) – BIL (cf. Fig. 8), (c) – DL (cf. Fig 10). Panels in each row display the total spectrum and its orientational and induced components. Color areas indicate standard deviations.

2. Snapshots of BIL for the positively and negatively charged UQ@H2O interfaces

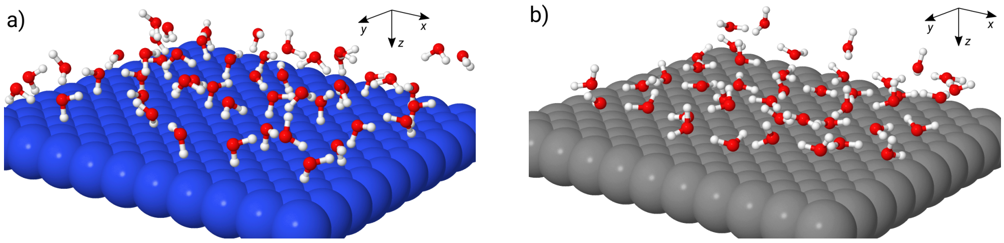

Figure S2. Snapshots of BIL region of the negatively (a) and positively (b) charged UQ@H2O interfaces.

3. Characteristics of positively charged solid/neat water interfaces

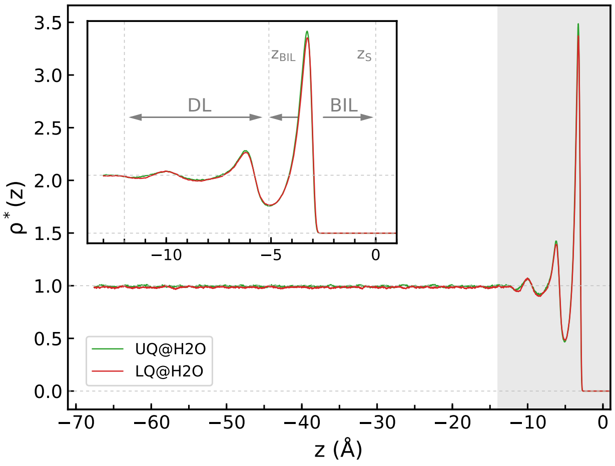

Figure S3. z-profile of water density $\rho(z)^*$ for the interfaces. The inset shows a zoom of the shaded area and indicates limits of the bonded interfacial layer (BIL) and diffuse layer (DL). The origin of the z-axis is at the position of surface atoms, z_S.

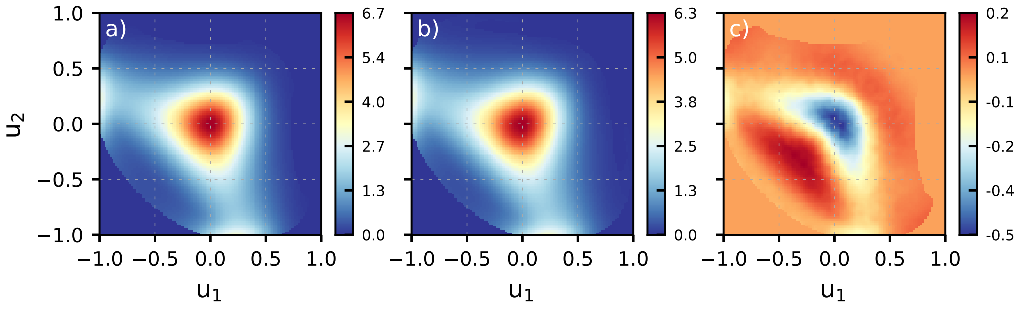

Figure S4. Maps of conditional probability density $\tilde{P}_{BIL}(u_1, u_2)$ of the interfaces. (a) – UQ@H2O, (b) – LQ@H2O, (c) – difference map (b) - (a).

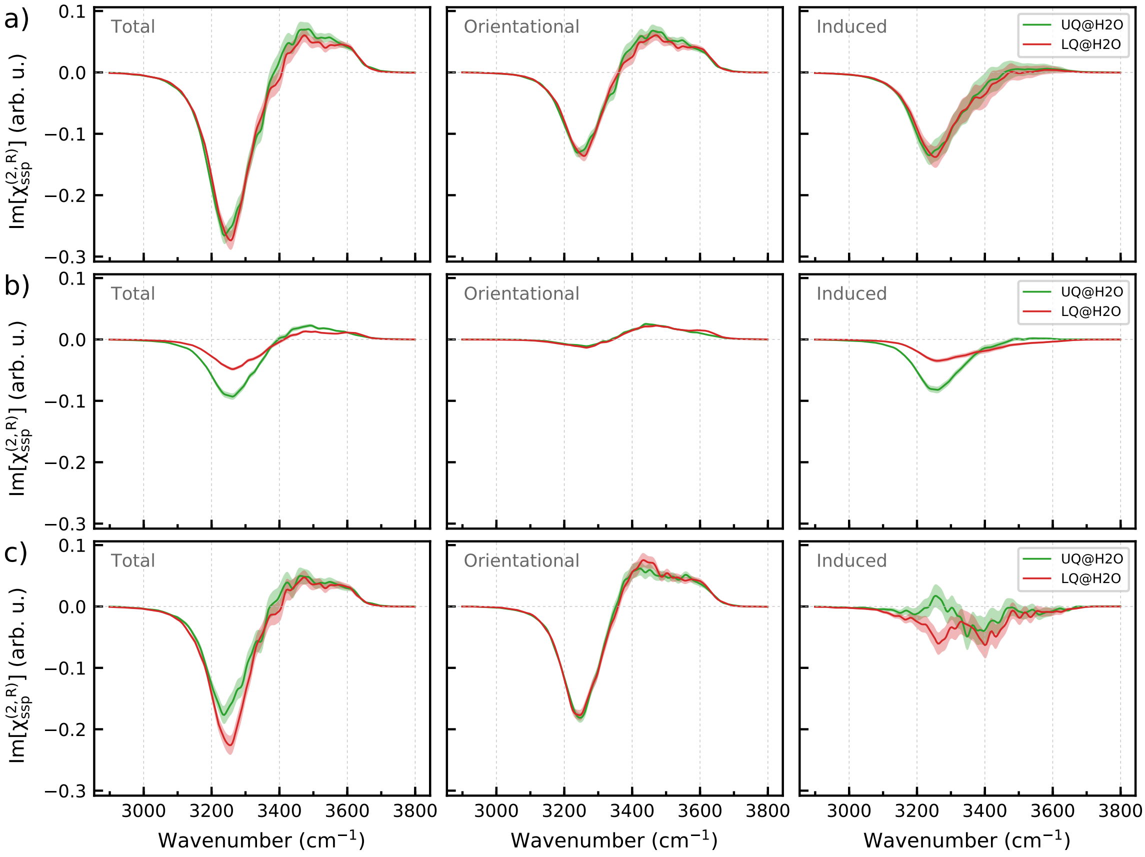

Figure S5. Breakdown of Im[$\chi^{(2)}_{ssp}$] spectra for the positively charged solid/water interfaces. (a) – spectra of a region from $z = z_{BIL}$ to $z = z_0$ (Fig. 1 of main article), (b) – BIL (cf. Fig. 8), (c) – DL (cf. Fig 10). Panels in each row display the total spectrum and its orientational and induced components. Color areas indicate standard deviations.
