# Supplementary figures and images for: Effects of Surface Charge Distribution and Electrolyte Ions on the Nonlinear Spectra of Model Solid–Water Interfaces
Source: Molecules. 2024 Aug 8;29(16):3758. doi: 10.3390/molecules29163758 (PMC11356812; doi:10.3390/molecules29163758)

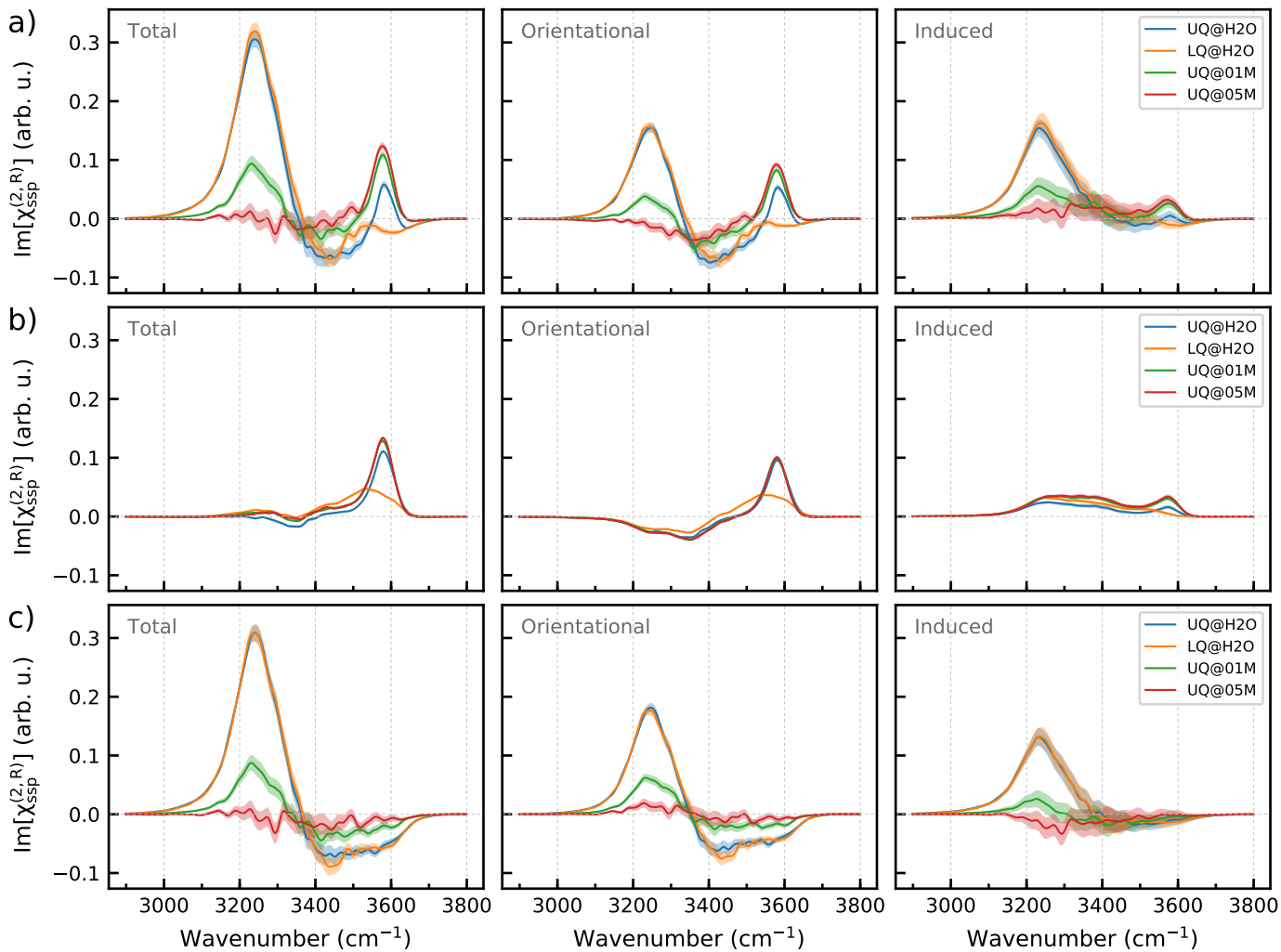

Supplement: Supplementary file 1 [file molecules-29-03758-s001.zip › chi2-decomposition-all-minus.pdf]

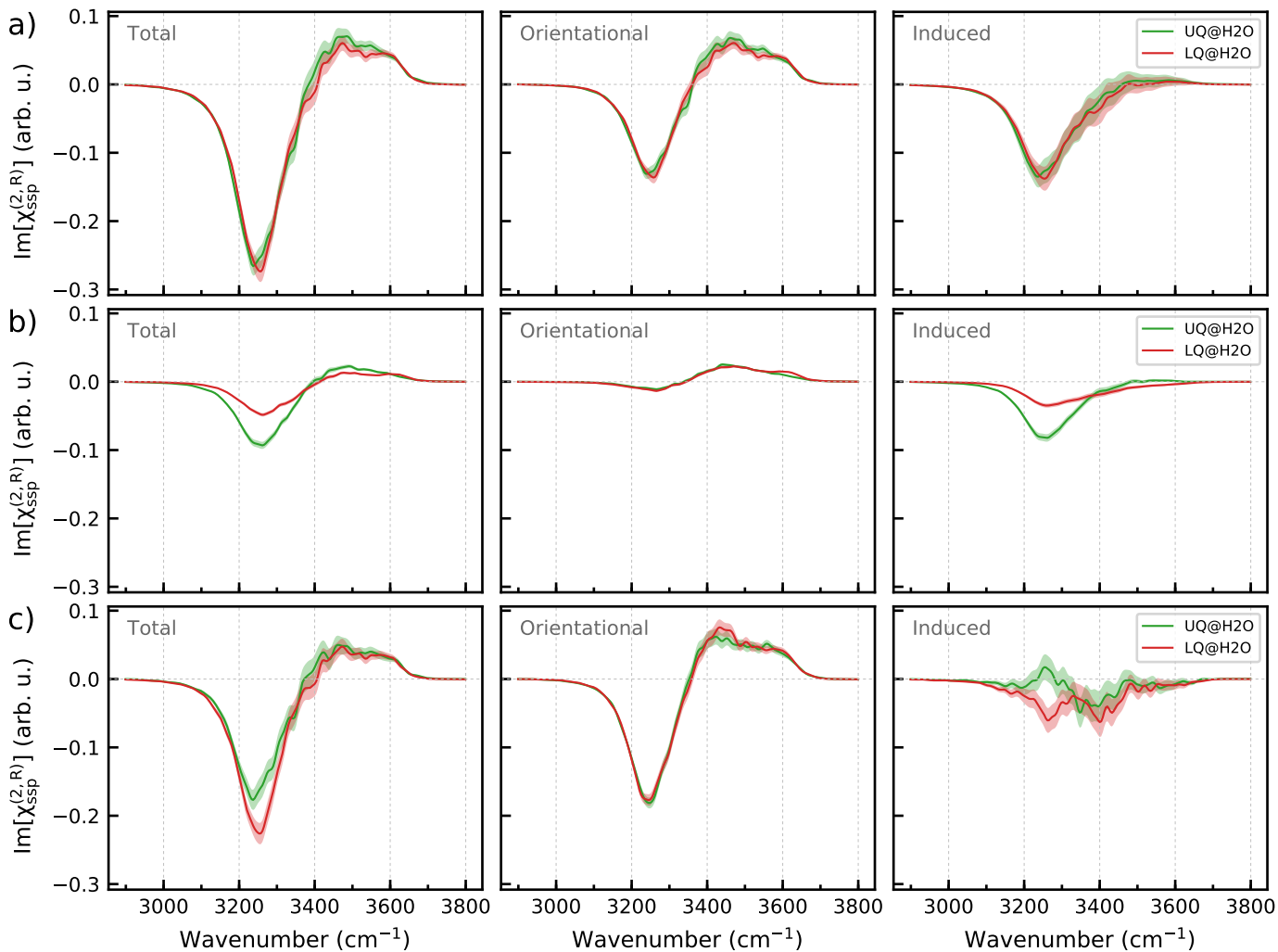

Supplement: Supplementary file 1 [file molecules-29-03758-s001.zip › chi2-decomposition-all-plus.pdf]

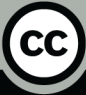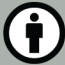

BY

Supplement: Supplementary file 1 [file molecules-29-03758-s001.zip › Definitions/logo-ccby-eps-converted-to.pdf]

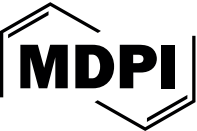

Supplement: Supplementary file 1 [file molecules-29-03758-s001.zip › Definitions/logo-mdpi-eps-converted-to.pdf]

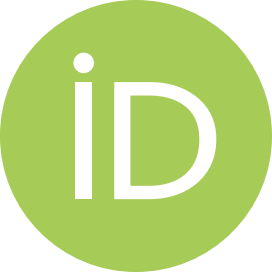

Supplement: Supplementary file 1 [file molecules-29-03758-s001.zip › Definitions/logo-orcid.pdf]

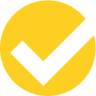

check for  
updates

Supplement: Supplementary file 1 [file molecules-29-03758-s001.zip › Definitions/logo-updates-eps-converted-to.pdf]

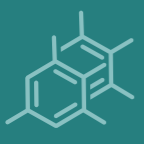

*molecules*

Supplement: Supplementary file 1 [file molecules-29-03758-s001.zip › Definitions/molecules-logo-eps-converted-to.pdf]

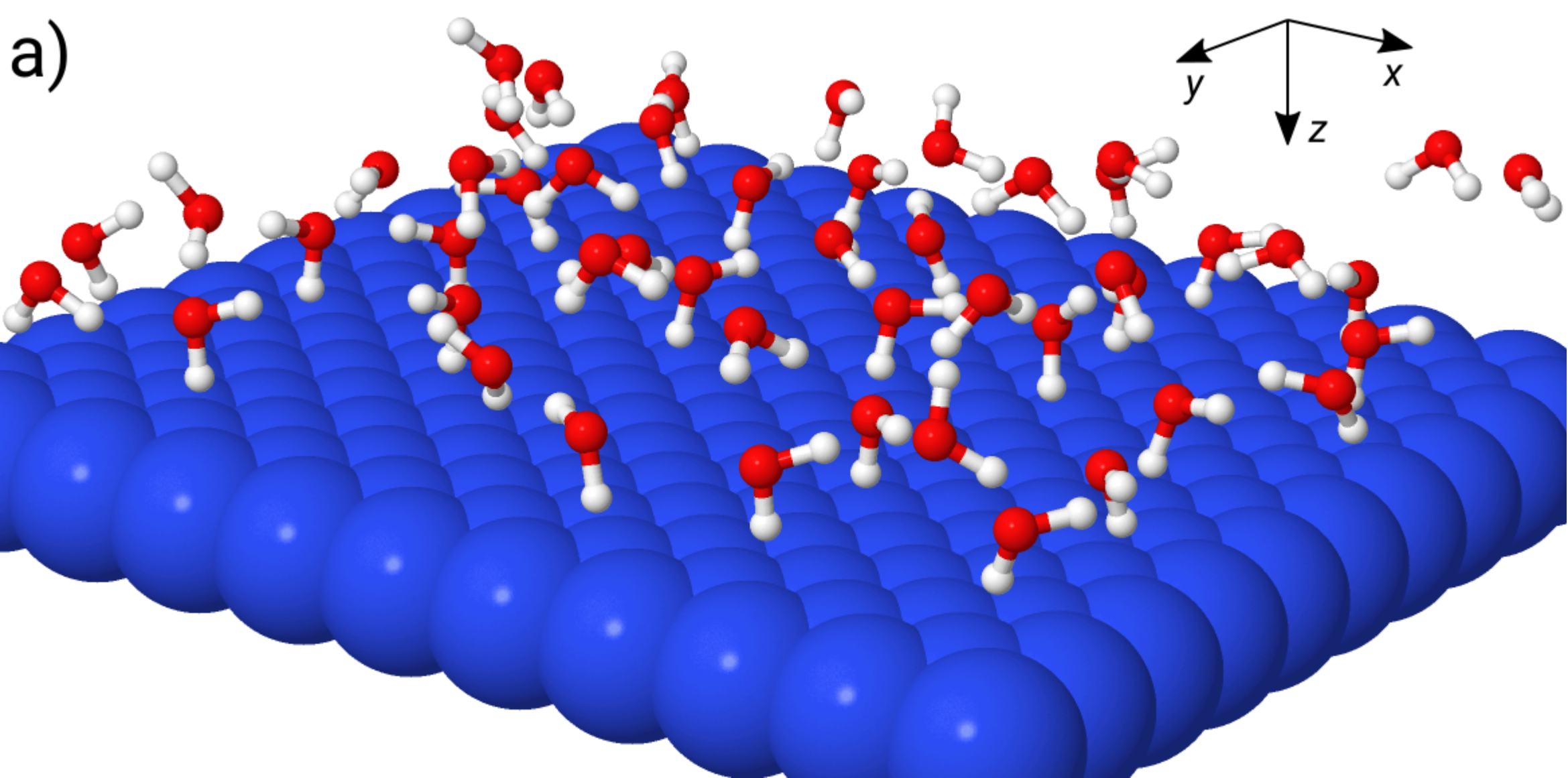

Supplement: Supplementary file 1 [file molecules-29-03758-s001.zip › n2-1.pdf]

b)

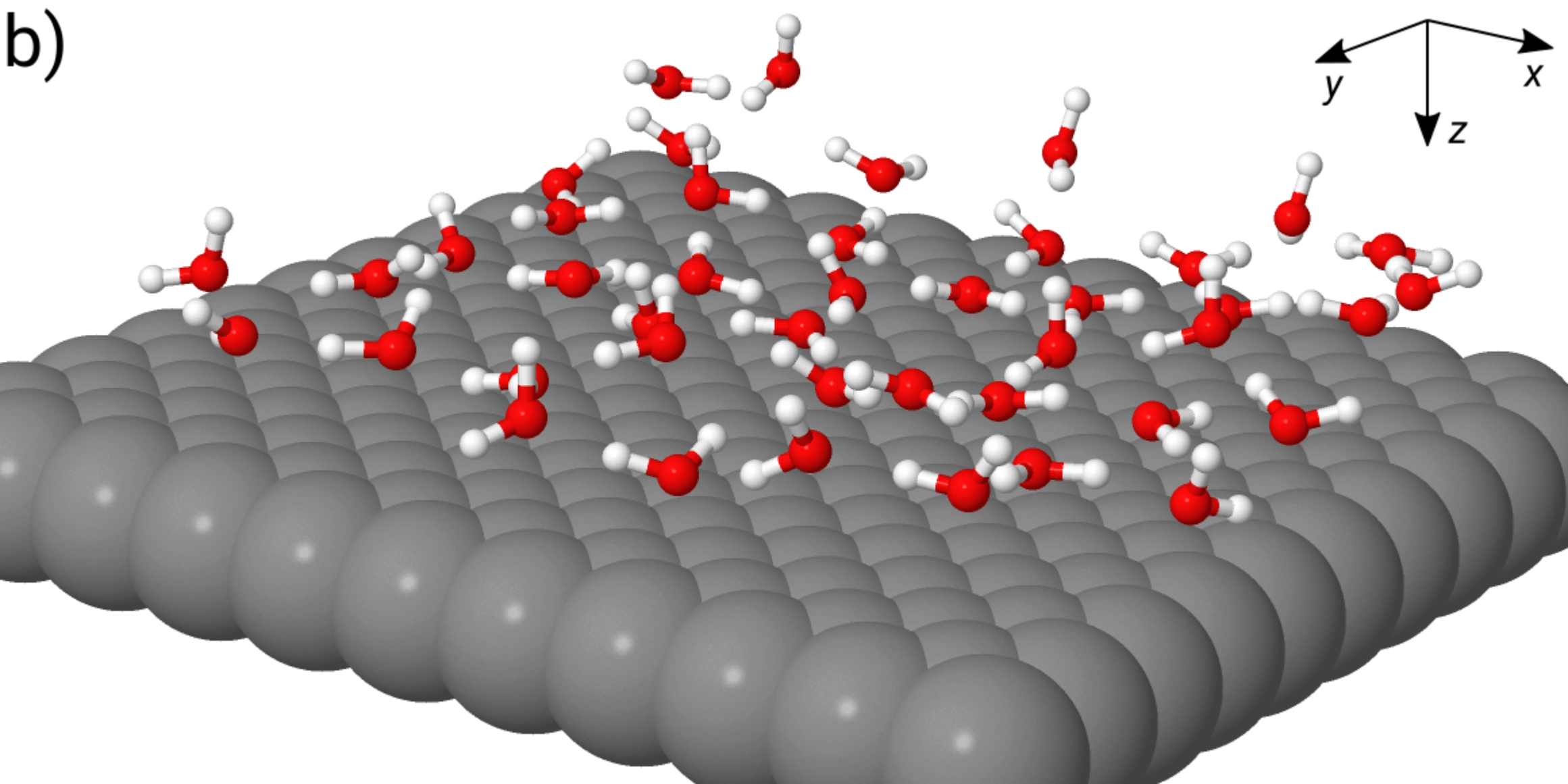

Supplement: Supplementary file 1 [file molecules-29-03758-s001.zip › p2-1.pdf]

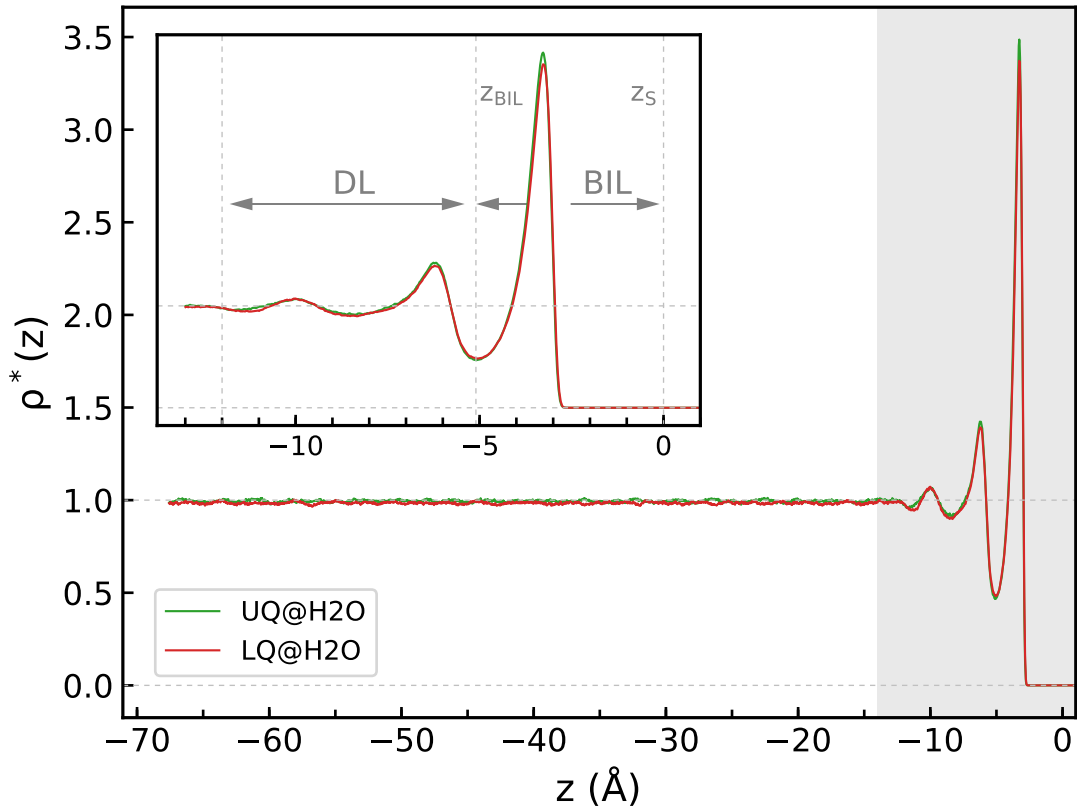

Supplement: Supplementary file 1 [file molecules-29-03758-s001.zip › rhoz-plot.pdf]

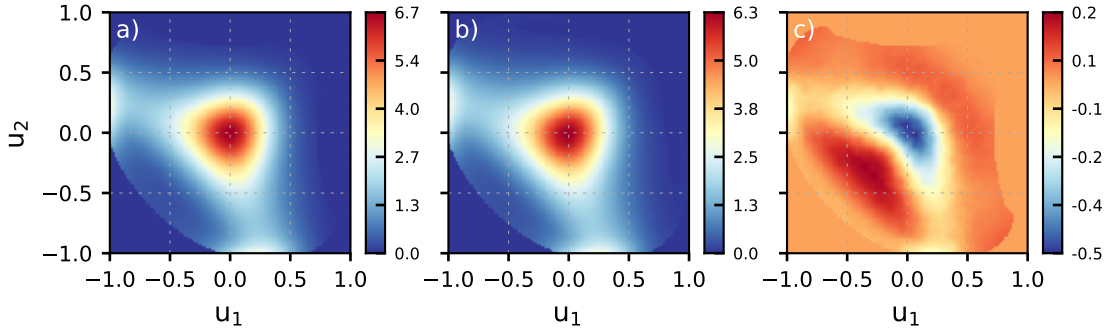

Supplement: Supplementary file 1 [file molecules-29-03758-s001.zip › zohmaps-bil.pdf]
